# Editorial: Advancements in antibody-based immunotherapy and cancer vaccines for hepatocellular carcinoma

**DOI:** 10.3389/fimmu.2025.1759881

**Published:** 2025-12-16

**Authors:** Ashraf A. Tabll, Anwaar Saeed, Mostafa S. Elkafrawy, Sherif A. El-Kafrawy

**Affiliations:** 1 Immunology Department, Egypt Center for Research and Regenerative Medicine (ECRRM), Cairo, Egypt; 2 Department of Medicine, Division of Hematology and Oncology, University of Pittsburgh Medical Center, Pittsburgh, PA, United States; 3 Faculty of Medicine, Menoufia University, Shebin El-Koam, Egypt; 4 Special Infectious Agents Unit–BioSafety Level 3 (BSL3), King Fahd Medical Research Center, King Abdulaziz University, Jeddah, Saudi Arabia; 5 Department of Medical Laboratory Sciences, Faculty of Applied Medical Sciences, King Abdulaziz University, Jeddah, Saudi Arabia

**Keywords:** antibody-based immunotherapy, cancer vaccine, checkpoint inhibitors, hepatocellular carcinoma, priority research gaps

## Introduction

Hepatocellular carcinoma (HCC) is a highly lethal malignancy with limited benefit from traditional therapies, especially in advanced disease. This Research Topic highlights recent advances in antibody-based immunotherapy and cancer vaccine development, addressing key challenges in biomarkers, combination strategies, safety, and mechanisms of response. Together, these studies advance precision immunotherapy for HCC while underscoring ongoing obstacles posed by tumor heterogeneity, immune suppression, and therapeutic resistance.

## Highlights from the Research Topic

### Additional clinical case reports: safety and toxicity signals

Quiles et al. describe a rare but severe case of atezolizumab-induced vanishing bile duct syndrome (VBDS) in a 63-year-old man who developed progressive cholestatic injury after three cycles of therapy. Biopsy showed loss of intrahepatic bile ducts in over half of portal tracts, and liver function failed to recover despite immunosuppression. This case highlights the need for early recognition of atypical immune-related adverse events, particularly cholestatic or bile-duct–centered injury patterns, and the importance of biopsy when biochemical abnormalities persist or do not respond to steroids.

Zhang et al. report fatal hepatorenal failure four days after starting tislelizumab plus anlotinib in a 72-year-old patient, likely reflecting synergistic toxicity, possibly exacerbated by concurrent infection. Together, these reports underscore the potential severity of ICI- and ICI/TKI-associated toxicity, emphasizing careful patient selection, vigilant monitoring, early histological assessment, and the urgent need for predictive biomarkers and more conservative escalation strategies in complex immunotherapy regimens.

### PD-L1 expression as a prognostic biomarker

Lee et al. examined PD-L1 expression in both malignant cells and tumor-infiltrating cells among 72 HCC patients treated with atezolizumab plus bevacizumab. Using Combined Positive Score (CPS) thresholds (CPS ≥10, 1–10, <1), they demonstrated that patients with CPS ≥10 had significantly improved overall survival (median OS 14.8 vs. 8.3 months; P = 0.046) and progression-free survival (median PFS 11 months; P = 0.044). Objective response rates were also highest in the CPS ≥10 group (53.3% vs. 27.3% and 16.7%). In multivariate analysis, PD-L1 expression ≥1 and ≥10 were independently associated with favorable prognosis.

Huai et al. explored the role of TEA domain transcription factor 1 (TEAD1) in liver hepatocellular carcinoma (LIHC). Their multi-omics analysis suggested that TEAD1 may serve as both a prognostic biomarker and an immunotherapeutic target, as it influences proliferation, invasion, and tumor immunology.

Beyond single biomarker validation, understanding the full spectrum of available antibody-based therapeutic modalities is essential for contextualizing these predictive findings within the broader treatment landscape.

### Antibody modalities in HCC immunotherapy

El-Kafrawy et al. contributed a thorough review of current and emerging antibody-based strategies for HCC, including monoclonal antibodies, bispecific antibodies, and antibody–drug conjugates, detailing mechanisms such as immune modulation, angiogenesis inhibition, and targeted cytotoxicity. They highlight breakthroughs like anti-PD-1/PD-L1 and CTLA-4 ICIs, along with approaches targeting glypican-3 (GPC3). The review emphasizes challenges, including tumor heterogeneity, resistance mechanisms, and immune-related adverse events, and advocates for strategic combination regimens and biomarker-driven selection to maximize therapeutic outcomes.

Translating this expanding therapeutic repertoire into evidence-based clinical practice requires systematic evaluation of comparative efficacy across treatment strategies, particularly for intermediate-stage HCC, where both locoregional and systemic approaches may be considered.

### Viral reactivation and immunotherapy

Xu et al. investigated hepatitis B virus (HBV) reactivation in patients with HBV-related HCC undergoing conversion therapy, which included hepatic artery infusion chemotherapy (HAIC), transarterial chemoembolization (TACE), tyrosine kinase inhibitors (TKIs), and ICIs. Their findings demonstrated that HBV reactivation was associated with reduced progression-free survival, emphasizing the importance of prophylactic antiviral therapy and rigorous HBV DNA monitoring in this clinical setting.

Having established the importance of safety monitoring in viral hepatitis contexts, several contributions in this Research Topic demonstrate how integrating locoregional interventions with systemic immunotherapy may enhance efficacy while managing treatment-related complications.

### Integration of locoregional and systemic therapies

Locoregional therapies continue to play an important role in HCC. Fang et al. systematically reviewed TACE combined with immune-targeted therapy in unresectable HCC. Their analysis showed that combination therapy offered superior local control and survival compared to ICIs alone, though at the expense of increased liver-related adverse events.

Chen et al. reported improved outcomes when TACE was combined with lenvatinib and tislelizumab in intermediate-stage HCC patients exceeding the up-to-11 criteria. Similarly, Li et al. compared PD-1 and PD-L1 inhibitors in combination with HAIC and lenvatinib, finding that PD-L1–based regimens yielded higher response rates with fewer severe adverse events.

While these clinical combination strategies demonstrate incremental benefits, breakthrough therapeutic advances may require more innovative high-order combinations that address multiple resistance mechanisms simultaneously.

### High-order combinatorial approaches

Dong et al. introduced an innovative high-order combination strategy integrating oncolytic herpes simplex virus (OHSV2-DSTEFAP5/CD3), glypican-3 (GPC3)-targeted CAR-T cells, and immunotoxins in preclinical models. This approach promoted immune activation and tumor microenvironment remodeling, leading to notable tumor regression and a 40% complete response rate in experimental models. These studies provide compelling evidence for combination strategies that exploit non-overlapping resistance mechanisms to enhance efficacy.

### Priority research gaps and future directions

Despite major progress, two research gaps require urgent attention. First, predictive biomarkers for immunotherapy response remain insufficient. Although candidates such as PD-L1 CPS ≥10, TEAD1, and HBV reactivation show promise, no validated markers reliably distinguish responders from patients with primary resistance driven by factors like Wnt/β-catenin signaling. Multimodal approaches integrating molecular profiling, liquid biopsies, radiomics, and immune-microenvironment analysis are essential for true precision immunotherapy. Second, HCC therapeutic vaccines remain early in development. Major challenges include identifying tumor-specific antigens, overcoming the liver’s highly immunosuppressive environment, and generating strong, durable CD8^+^ T-cell responses in patients with cirrhosis or chronic viral infection.

### Call to action

We urge clinicians to prioritize systematic biospecimen collection (tumor tissue, normal liver, serial blood, and archival samples) with standardized annotation and longitudinal follow-up to strengthen real-world evidence. Researchers should focus on three priorities: developing composite biomarker panels through multicenter collaboration; defining resistance mechanisms such as Wnt/β-catenin, metabolic rewiring, and myeloid checkpoints to guide rational combinations; and advancing therapeutic vaccine platforms using organoids and humanized models to identify HCC-specific neoantigens and optimize strategies targeting antigens such as GPC3. Progress will require global cooperation, data sharing, translationally oriented studies, and adaptive trial designs to accelerate breakthroughs toward curative immunotherapy.

